# Role of PI3K/Akt and MEK/ERK Signalling in cAMP/Epac-Mediated Endothelial Barrier Stabilisation

**DOI:** 10.3389/fphys.2019.01387

**Published:** 2019-11-07

**Authors:** Dursun Gündüz, Christian Troidl, Christian Tanislav, Susanne Rohrbach, Christian Hamm, Muhammad Aslam

**Affiliations:** ^1^Department of Cardiology and Angiology, University Hospital of Giessen and Marburg, Giessen, Germany; ^2^Department of Cardiology and Angiology, Evangelisches Jung Stilling Krankenhaus GmbH, Siegen, Germany; ^3^Experimental Cardiology, Justus Liebig University Giessen, Giessen, Germany; ^4^Department of Neurology, Evangelisches Jung Stilling Krankenhaus GmbH, Siegen, Germany; ^5^Department of Neurology, University Hospital of Giessen and Marburg, Giessen, Germany; ^6^Institute of Physiology, Justus Liebig University Giessen, Giessen, Germany

**Keywords:** adherens junctions, Rac1, peripheral actin, cell survival, VE-cadherin, endothelial permeability, MEK/ERK, PI3K/Akt

## Abstract

**Background and Aims:**

Activation of the cAMP/Epac signalling stabilises endothelial barrier function. Moreover, its activation is accompanied by an activation of PI3K/Akt and MEK/ERK signalling in diverse cell types but their impact on endothelial barrier function is largely unknown. Here the role of PI3K/Akt and MEK/ERK signalling in cAMP/Epac-mediated endothelial barrier stabilisation was analysed.

**Methods:**

Endothelial barrier function was analysed in cultured human umbilical vein endothelial cells (HUVECs) by measuring flux of albumin. A modified cAMP analogue 8-pCPT-2′-O-Me-cAMP (Epac agonist) was used to specifically activate cAMP/Epac signalling.

**Results:**

Epac agonist reduces the basal and attenuates thrombin-induced endothelial hyperpermeability accompanied by an activation of PI3K/Akt and MEK/ERK signalling. The qPCR data demonstrate HUVECs express PI3Kα, PI3Kβ, and PI3Kγ but not PI3Kδ isoforms. The western blot data demonstrate Epac agonist activates PI3Kα and PI3Kβ isoforms. Inhibition of MEK/ERK but not PI3K/Akt pathway potentiates the endothelial barrier protective effects of cAMP/Epac signalling. Inhibition of MEK/ERK signalling in the presence of Epac agonist induces a reorganisation of actin cytoskeleton to the cell periphery, enhanced VE-cadherin localisation at cell-cell junctions, and dephosphorylation of myosin light chains (MLC) but not inhibition of RhoA/Rock signalling. Moreover, Epac agonist promotes endothelial cell (EC) survival via reduction in activities of pro-apoptotic caspases in a PI3K/Akt and MEK/ERK signalling-dependent manner.

**Conclusion:**

Our data demonstrate that the Epac agonist simultaneously activates diverse signalling pathways in ECs, which may have differential effects on endothelial barrier function. It activates PI3K/Akt and MEK/ERK signalling which mainly govern its pro-survival effects on ECs. Inhibition of MEK/ERK but not PI3K/Akt signalling enhances barrier stabilising and barrier protective effects of cAMP/Epac activation.

**Chemical Compounds Used In This Study:**

8-pCPT-2′-O-Me-cAMP (PubChem CID: 9913268); Akt inhibitor VIII (PubChem CID: 10196499); AS-252424 (PubChem CID: 11630874); IC-87114 (PubChem CID: 9908783); PD 98059 (PubChem CID: 4713); PIK-75 (PubChem CID: 10275789); TGX-221 (PubChem CID: 9907093); Thrombin (PubChem CID: 90470996); U0126 (PubChem CID: 3006531); Wortmannin (PubChem CID: 312145).

## Introduction

Endothelial cell (EC) monolayer forms a selective semi-permeable barrier regulating the trafficking of macromolecules and blood cells across the vessel wall (Mehta and Malik, 2006). The major regulators of EC barrier function are the actin-myosin based EC contractile machinery (Garcia et al., 1995) and actin cytoskeleton-anchored adherens junctions (AJs) consisting of VE-cadherin and catenins linked to the actin cytoskeleton (Dejana et al., 2008). Intracellular cAMP levels play an important role in the maintenance and restoration of basal EC barrier function (Baumer et al., 2009; Aslam et al., 2014). Endothelial barrier function is also modulated by the contractile forces generated by the actin-myosin-based contractile machinery (Schnittler et al., 1990). The activation of the contractile machinery is controlled by the phosphorylation state of regulatory myosin light chains (MLC) that is induced by myosin light chain kinase (MLCK) (Wysolmerski and Lagunoff, 1991) and dephosphorylated by myosin light chain phosphatase (MLCP) (Goeckeler and Wysolmerski, 2005). Activation of Rho/Rho kinase (Rock) and MEK/ERK pathways has been demonstrated to induce MLC phosphorylation via inhibition of MLCP or activation of MLCK, respectively (Nguyen et al., 1999; Birukova et al., 2004). Thrombin inhibits MLCP via increased phosphorylation of regulatory subunit MYPT1 at T850 and activates MLCK in Ca^2+^/calmodulin-dependent manner (Goeckeler and Wysolmerski, 1995; Birukova et al., 2004) that contribute to its EC barrier destabilisation properties.

The mechanisms increasing intracellular levels of cAMP protect EC barrier against a variety of inflammatory stimuli (van Hinsbergh and van Nieuw Amerongen, 2002; Aslam et al., 2010). Cellular cAMP downstream activates two different signalling pathways via its effectors, (1) the classical PKA and (2) the relatively new Epac (exchange protein directly activated by cAMP) signalling (de Rooij et al., 1998). We and others have previously demonstrated that activation of both pathways stabilises EC barrier function via differential mechanisms (Cullere et al., 2005; Lorenowicz et al., 2008; Aslam et al., 2010) and both pathways converge at the Rac1-mediated actin cytoskeleton remodelling (Aslam et al., 2014; Schlegel and Waschke, 2014). Although both PKA and Epac agonists induce Rac1-mediated actin remodelling, activation of PKA but not Epac inhibits RhoA activity and downstream EC contractile machinery (Aslam et al., 2010).

In addition to Rac1, the cAMP/Epac pathway activates PI3K/Akt signalling in ECs (Namkoong et al., 2009) and MEK/ERK signalling in ECs (Hashimoto et al., 2015) and non-ECs (Fu et al., 2011). In the present study we further investigated and analysed the role of these pathways and their possible interaction with cAMP/Epac-mediated EC barrier stabilising effect. The cAMP/Epac signalling was activated by using a modified cAMP analogue, 8-pCPT-2′-O-Me-cAMP (8-CPT-cAMP), which preferentially targets Epac but not PKA (Aslam et al., 2010). The study was conducted on a well-established *in vitro* cell-culture model of human umbilical vein ECs (HUVECs). We demonstrate that the Epac agonist 8-CPT-cAMP activates PI3Kα and PI3Kβ signalling which regulates the EC survival but not its EC barrier stabilising actions. Moreover, it also activates MEK/ERK signalling and pharmacological inhibition of which enhances the Epac agonist-mediated EC barrier stabilising effects.

## Materials and Methods

### Materials

Anti Ki67 antibody was from Abcam (Cambridge, United Kingdom); HRP-conjugated anti-mouse IgG and -rabbit IgG antibodies were from Amersham Biosciences (Heidelberg, Germany); anti VE-cadherin was from Beckman Coulter (Krefeld, Germany); 8-pCPT-2′-O-Me-cAMP (8-CPT-cAMP) was from Biolog (Bremen, Germany); anti phospho Akt (Ser473), anti phospho ERK, anti phospho MLC (S18/T19), anti phospho MYPT1 (T850), and anti GAPDH were from Cell Signaling Technologies (Danvers, MA, United States); Pierce^®^ ECL solution was from Thermo Fischer Scientific (Niederlassung Nidderau, Germany); Complete^®^ protease inhibitor cocktail was from Roche (Mannheim, Germany); ThinCert^®^ polycarbonate membrane filters (6-well) were from Greiner Bio-One (Frickenhausen, Germany); Benzonase^®^ anti phospho MYPT1 (Thr850), and MEK inhibitor U0126 were from Merck-Millipore (Darmstadt, Germany); EC basal medium plus supplement pack was from PromoCell (Heidelberg, Germany); isoform specific PI3K inhibitors PIK-75, TGX-221, AS-252424, and IC-87114 were from Selleckchem (Boston, MA, United States); Akt inhibitor VIII, human thrombin, phalloidin-TRITC, and Wortmannin were from Sigma (Steinheim, Germany). All other chemicals were of the best available quality, usually analytical grade.

### Cell Culture

The study conforms to the principles outlined in the “Declaration of Helsinki” (doi: 10.1016/S0008-6363(97)00108-9). HUVEC were isolated and cultured as described previously (Aslam et al., 2012) in complete EC culture medium (Cat # C-22010; PromoCell, Heidelberg, Germany) and used at passage 1–2. HUVEC were cultured in 6-well plates for western blotting, 6-well filter inserts for permeability assay, 96-well plates for caspase 3/7 activity, 12-well plates for cell counting assay, and 10 cm culture dishes for pulldown assay.

### Experimental Protocols

The basal medium used in incubations was modified Tyrode’s solution (composition in mM: 150 NaCl, 2.7 KCl, 1.2 KH_2_PO_4_, 1.2 MgSO_4_, 1.0 CaCl_2_, and 30.0 *N*-2-hydroxyethylpiperazine-*N*′-2-ethanesulfonic acid; pH 7.4, 37°C). Agents were added as indicated. Stock solutions of 8-CPT-cAMP and thrombin were prepared in basal medium and that of Akt inhibitor VIII, PIK-75, TGX-221, AS-252424, IC-87114, U0126, and Wortmannin in DMSO. Appropriate volumes of these solutions were added to the cells yielding final solvent concentrations ≤0.1% (vol/vol). Where combination of drugs was used, inhibitors were added 30–60 min before adding the 8-CPT-cAMP. The same final concentrations of basal medium and DMSO were included in all respective control experiments.

In a set of pilot experiments the concentration-response relationships were determined to find the optimal concentration of the drugs used in the study. The drugs were used at following final concentrations; thrombin (0.3 IU/ml), 8-CPT-cAMP (200 μM), U0126 (10 μM), Akt-inhibitor VIII (5 μM), PIK-75 (0.1 μM), TGX-221 (1 μM), AS-252424 (1 μM), IC-87114 (1 μM), and Wortmannin (0.1 μM).

### Immunocytochemistry and Confocal Microscopy

Immunocytochemistry and confocal microscopy was performed as described previously (Aslam et al., 2014). Briefly, HUVEC were grown until confluence on glass cover slips. After treatment cells were fixed with 4% PFA, permeabilised with 0.2% Triton X-100, and blocked with blocking solution (5% BSA + 5% FCS) for 1 h. Cells were incubated with the primary antibody overnight at 4°C and with the secondary antibody for 1 h at RT. For actin cells were stained with phalloidin-TRITC (1:50) for 1 h at RT. The cover slips were embedded in fluorescent mounting medium (CitiFluor, United Kingdom) and put onto glass slides. Images were obtained using a Zeiss LSM 510 META (Zeiss; Jena, Germany) confocal microscope.

### Macromolecule Permeability Measurement

The permeability of trypan-blue-labelled albumin across HUVEC monolayers was analysed as previously described (Aslam et al., 2012).

### Western Blotting

Western blotting was performed as described previously (Aslam et al., 2012). Blots were imaged using Fusion-FX7 imager (VWR Erlangen, Germany) and unsaturated images were analysed using Quantity-One software (Bio-Rad, Germany). GAPDH from same gel was used as loading control for normalisation of the respective protein signal.

### Rac1 Pulldown Assay

Rac1 pulldown assay was performed using commercial kit (Thermo Fischer Scientific, Germany) as described recently (Aslam et al., 2014). The pulled-down Rac1 protein bands (Rac1-GTP) were normalised with Rac1 bands from respective total cell lysates (Rac1-total).

### Cell Number

Cell number was measured as described recently (Gündüz et al., 2015). *Briefly*, HUVECs were seeded at a density of 5 × 10^4^/well in 12-well cell culture plates. Cell cycle arrest was performed by incubating cells in growth factor free low serum medium for 24 h. The cells were then incubated with DMSO, Akt or MEK inhibitors for 1 h, replaced the medium with without inhibitors and then treated with 8-CPT for 24 h low serum and reduced growth factor medium. The cells were then trypsinised and counted manually using a Neubauer cell chamber.

### Caspase 3/7 Activity

Caspase 3/7 activity was measured using commercial Caspase-Glo assay kit from Promega (Madison, WI, United States) as described recently (Gündüz et al., 2015). The fluorescence was measured using Infinite 200^®^ ELISA reader (Tecan, Austria). In order to avoid the masking effects of growth factors present in the complete EC culture medium, these sets of experiments were performed under reduced growth factors and serum conditions.

### RNA Isolation and PCR

Total RNA was isolated using RNeasy mini kit (Qiagen, Hilden, Germany) and genomic DNA was removed by treatment with DNase (Qiagen, Hilden, Germany). Total RNA (100 ng) was used in a 20 μl reverse transcriptase reaction to synthesize cDNA using iScript cDNA synthesis kit (Bio-Rad Inc., Hercules, CA, United States). RT reactions were performed for 30 min at 42°C. PCR reactions were performed using Bio-rad CFX96 real time system (Bio-Rad Inc., Hercules, CA, United States) using SYBR green master mix from Bimake (Houston, TX, United States). The thermal cycling program consisted of one denaturation cycle of 5 min at 95°C followed by 40 cycles each of 30 s at 95°C, 30 s at 60°C, and 30 s at 72°C. The primer sequences used are listed in Table 1. The PCR products were separated by electrophoresis on a 1% Tris-acetate-EDTA agarose gel.

**TABLE 1:** Primer sequences used in the study.

**Gene**	**Locus ID**	**Forward primer (5**′**–3**′)	**Reverse primer (5**′**–3**′)	**Fragment (bp)**
PI3Kα	NM_006218.3	ggacccgatgcggttagag	atcaagtggatgccccacag	168
PI3Kβ	NM_006219.2	ccttcgataagagtcgaggtgg	gcagtcttgtcgcaaagtcc	125
PI3Kγ	NM_002649.3	actgaagaaaagtttcaggcagc	ccaagaatgtgcccgaagtc	161
PI3Kδ	NM_005026.3	ctgcgccgggacgataag	ccagaattccatggggcagt	186
GAPDH	NM_002046.5	tgcaccaccaactgcttagc	ggcatggactgtggtcatgag	87

### Statistical Analysis

The data are presented as means (±SEM) of 3–5 experiments from independent cell preparations. The comparison between multiple groups was performed by one-way analysis of variance (ANOVA) followed by a Student–Newman–Keuls *post hoc* test using GraphPad Prism 6 software (GraphPad Inc., San Diego, CA, United States). The “*P*” values of ≤0.05 were considered statistically significant.

## Results

### Epac Agonist Phosphorylates Akt via PI3Kα and PI3Kβ

**FIGURE 1:**
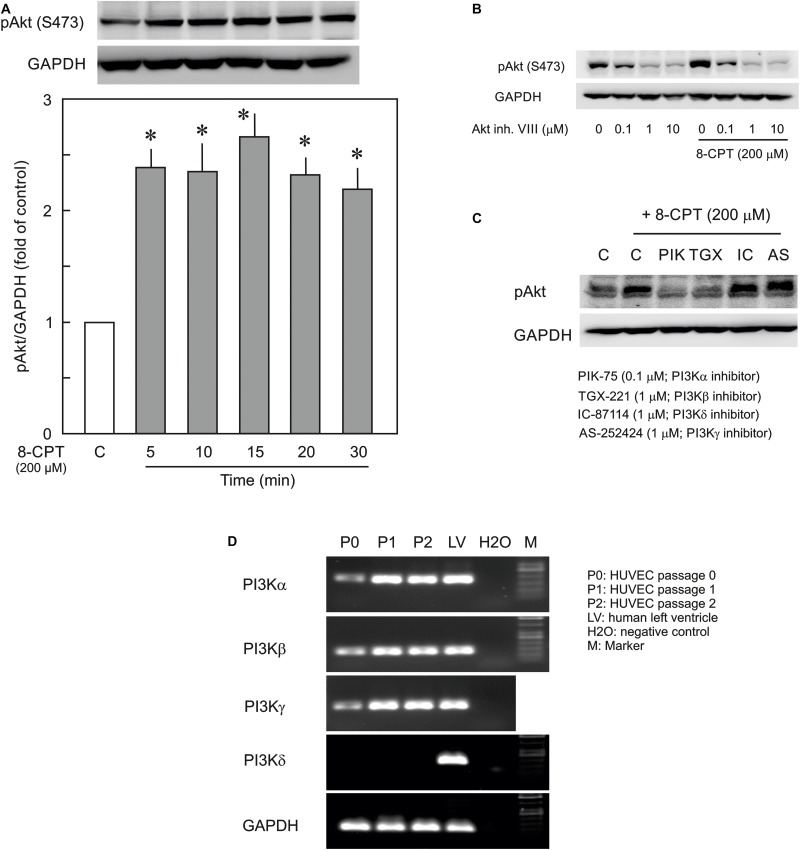
Effect of the Epac agonist on PI3K/Akt signalling in HUVECs. **(A)** Time-dependent effect of the Epac agonist 8-CPT-cAMP (8-CPT) on Akt phosphorylation in ECs. *Upper panel:* representative blots of Akt phosphorylation at S473. *Lower panels:* densitometric analysis of western blots. *n* = 3; *P* < 0.05 for all subfigures; ^∗^ vs. control. **(B)** Effect of Akt inhibitor VIII (Akt inh. VIII) on the Epac agonist 8-CPT-induced Akt phosphorylation. ECs were incubated with different concentrations of Akt inh. VIII as indicated for 30 min and were exposed to 8-CPT for 20 min. **(C)** ECs were pre-treated with isoform specific PI3K inhibitors or DMSO as indicated before adding 8-CPT for 20 min. and Akt phosphorylation at S473 was analysed by western blot. GAPDH from the same blot was used as loading control. Representative blots from three different experiments. **(D)** The mRNA expression of PI3K isoforms in HUVEC (Freshly isolated cells, P0; Passage 1, P1; and Passage 2, P2). RNA from human left ventricular tissue (LV) was used as positive control.

In the first instance the activation of PI3K/Akt signalling by the Epac agonist, 8-CPT-cAMP, in ECs was investigated by determining the phosphorylation state of Akt at Ser473. The Epac agonist, 8-CPT-cAMP (200 μM) caused a ∼2.5-fold increase in Akt phosphorylation within 5 min which persisted during the whole time of measurement (30 min) demonstrating a strong activation of PI3K/Akt signalling pathway (Figure 1A). The similar level of activation was also achieved using lower concentration (100 μM) of the agonist (Supplementary Figure S1). The Epac agonist-induced Akt phosphorylation was abrogated completely by specific pharmacological inhibitors of Akt, PI3Kα, and PI3Kβ but not PI3Kγ and PI3Kδ (Figures 1B,C). In order to investigate the expression of PI3K isoforms in ECs we performed qPCR using total RNA from primary HUVEC (passages 0–2). As shown in Figure 1D, HUVEC express PI3Kα, PI3Kβ, and PI3Kγ but not PI3Kδ. Human left ventricle tissue was used as positive control which expresses all PI3K isoforms. Interestingly the expression of all expressed isoforms of PI3K was relatively low in primary (passage 0) HUVECs, however, their expression increased on passaging (Figure 1D and Table 2).

**TABLE 2:** Relative expression of PI3K isoforms in HUVEC (passage 0–2).

**C_T_**	**GAPDH**	**PI3Kα**	**PI3Kβ**	**PI3Kγ**	**PI3Kδ**
Primary	23.1	35.1	36.0	35.0	−
Passage 1	20.4	30.8	31.9	29.3	−
Passage 2	21.0	31.7	32.9	29.0	−
hLV	17.8	28.0	29.5	28.6	29.5

**ΔC_T_**	**PI3Kα**	**PI3Kβ**	**PI3Kγ**	**PI3Kδ**	

Primary	12.0	12.9	11.9	−	
Passage 1	10.4	11.5	8.9	−	
Passage 2	10.7	11.9	8.0	−	
hLV	10.2	11.7	10.8	11.7	

### Role of PI3K/Akt Signalling in cAMP/Epac-Mediated EC Barrier Stabilisation

**FIGURE 2:**
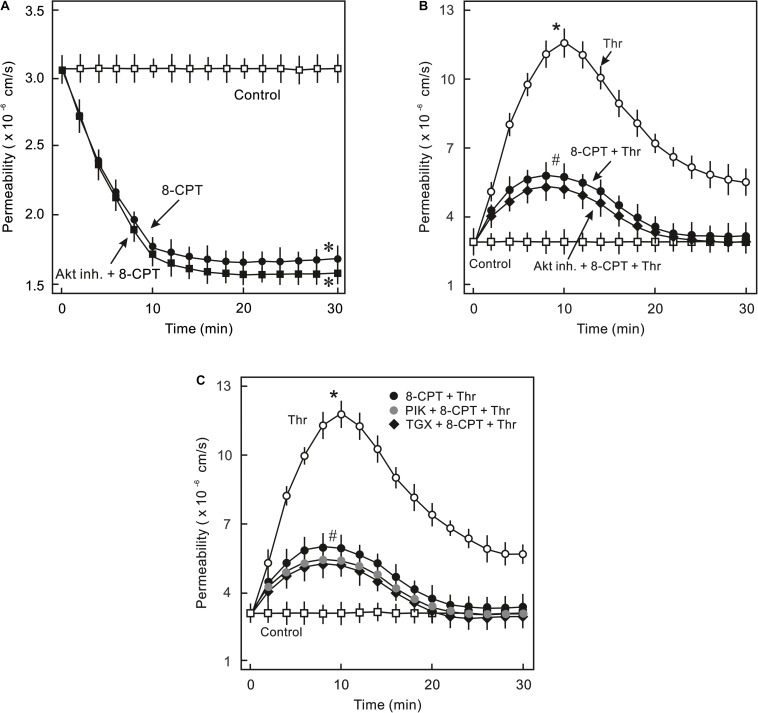
Analysis of role of PI3K/Akt signalling in Epac-mediated EC barrier stabilisation. **(A)** Effect of Akt inhibition on Epac-mediated EC barrier stabilisation. ECs were pre-incubated with Akt inh. VIII (5 μM) or DMSO for 30 min before adding the Epac agonist 8-CPT (200 μM) and albumin permeability was measured. ^∗^ vs. control. **(B)** EC were pre-incubated with Akt inh. VIII (5 μM) or DMSO for 30 min before adding Epac agonist 8-CPT (200 μM) and thrombin (Thr; 0.3 IU/ml). ^∗^ vs. control; # vs. Thr alone; n.s.: not significantly different. **(C)** EC were pre-incubated with isoform specific PI3K inhibitors; PI3Kα (PIK; 0.1 μM) and PI3Kβ (TGX; 1 μM) or DMSO for 30 min before adding the Epac agonist 8-CPT (200 μM) and Thr (0.3 IU/ml). ^∗^ vs. control; # vs. Thr alone.

**FIGURE 3:**
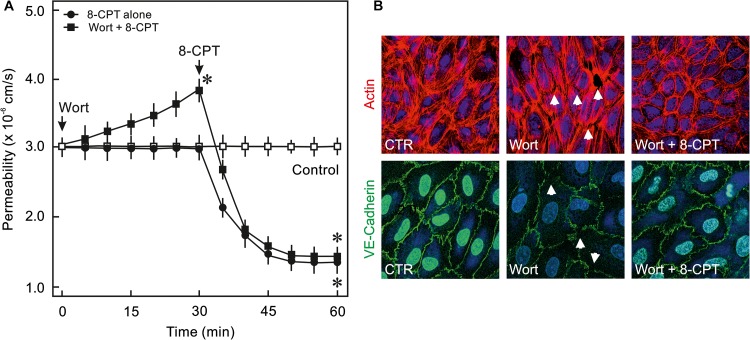
Effect of PI3K inhibitor Wortmannin on EC barrier function. **(A)** ECs were pre-incubated with Wortmannin (0.1 μM) or DMSO for 30 min before adding the Epac agonist 8-CPT (200 μM) and albumin permeability was measured. The arrows indicate when the respective drugs were added. ^∗^ vs. control. **(B)** Effect of the Epac agonist on EC actin cytoskeleton and AJs. Representative immunofluorescence images of F-actin labelled with phalloidin-TRITC and VE-cadherin from three experiments of independent cell preparation. The nuclei were stained with DAPI.

In order to analyse whether PI3K/Akt signalling plays a role in cAMP/Epac-mediated EC barrier stabilisation pharmacological inhibitors of Akt and PI3K were employed to specifically inhibit Akt or PI3K isoform. As shown in Figures 2A,B, 8-CPT-cAMP reduced basal as well as thrombin-induced hyperpermeability. Inhibition of Akt using Akt inhibitor VIII neither antagonised 8-CPT-cAMP-mediated reduction in basal EC permeability (Figure 2A) nor abrogated its protective effect against thrombin-induced hyperpermeability (Figure 2B). Similarly, pharmacological inhibition of PI3Kα and PI3Kβ had no effect on Epac-mediated EC barrier protection (Figure 2C). In contrast to these data, Wortmannin, a non-specific PI3K inhibitor, induced an increase in basal EC permeability and loss of cell-cell junctions which was completely abrogated by the Epac agonist (Figure 3). These data suggests that Wortmannin inhibits non-specifically other kinases required for EC barrier function and thus induces EC hyperpermeability.

### Epac Agonist Activates MEK/ERK MAPK Signalling in ECs

**FIGURE 4:**
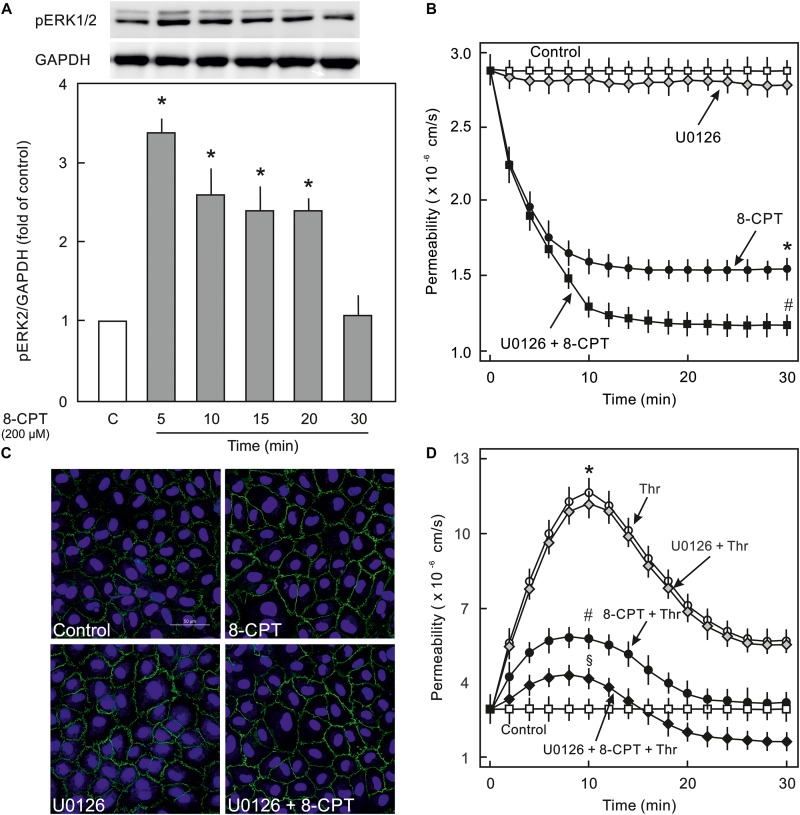
Effect of Epac activation on MEK/ERK signalling. **(A)** Time-dependent effect of the Epac agonist 8-CPT-cAMP (8-CPT) on ERK phosphorylation in ECs. *Upper panel:* Representative blots of ERK phosphorylation. *Lower panels:* Densitometric analysis of western blots. *n* = 3; *P* < 0.05 for all subfigures; ^∗^ vs. control. **(B)** Effect of MEK/ERK inhibition on Epac-mediated EC barrier stabilisation. EC were pre-incubated with U0126 (10 μM) or DMSO for 30 min before adding the Epac agonist 8-CPT (200 μM) and albumin permeability was measured. ^∗^ vs. control; # vs. 8-CPT alone. **(C)** Effect of MEK-inhibition on Epac-mediated EC AJs stabilisation. ECs were treated with agents as indicated. Where inhibitors were used, these were added 30 min before adding the agonist. Representative immunofluorescence images of VE-cadherin from three experiments of independent cell preparation. The nuclei were stained with DAPI. **(D)** EC were pre-incubated with MEK inhibitor (10 μM) or DMSO before adding the Epac agonist 8-CPT (200 μM) and thrombin (Thr; 0.3 IU/ml). ^∗^ vs. control; # vs. Thr alone; §vs. 8-CPT + Thr.

Epac activation in non-ECs induces an activation of MEK/ERK (p44/p42 MAPK) signalling (Fu et al., 2011), therefore, it was analysed whether cAMP/Epac signalling also activates MEK/ERK signalling in ECs and if it plays a role in cAMP/Epac-mediated EC barrier stabilisation. As shown in Figure 4A and Supplementary Figure S2 the Epac agonist induced a transient but strong phosphorylation of ERK which returned to basal level within 30 min. In order to analyse the role of MEK/ERK signalling in Epac-mediated EC barrier stabilisation, MAPK signalling was inhibited pharmacologically using U0126, a highly specific pharmacological inhibitor of MEK. Inhibition of MEK/ERK itself had no effect on basal EC permeability (Figure 4B) as well as thrombin-induced hyperpermeability (Figure 4D) and VE-cadherin localisation at cell-cell junctions (Figure 4C). However, MEK inhibitor enhanced the permeability-reducing effect of the Epac agonist (Figure 4B) accompanied by an enhanced localisation of VE-cadherin at cell-cell junctions (Figure 4C). Similarly, the Epac agonist-mediated EC barrier protection against thrombin-induced hyperpermeability was enhanced in the presence of MEK inhibitor (Figure 4D).

### MEK/ERK Signalling and EC Actin Cytoskeleton and Contractile Machinery

**FIGURE 5:**
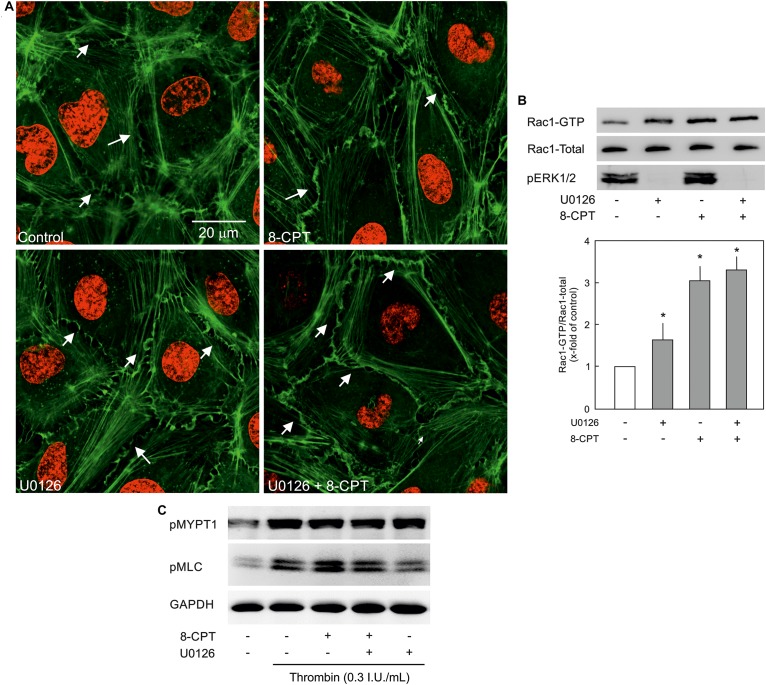
Effect of MEK/ERK inhibition on actin cytoskeleton remodelling. **(A)** Effect of MEK/ERK inhibition on actin cytoskeleton. Representative immunofluorescence images of actin cytoskeleton stained with phalloidin-TRITC (Green) from three experiments of independent cell preparation. Arrows indicate peripheral actin (Scale bar = 20 μm). Nuclei were stained with DAPI (Red). **(B)** Effect of MEK inhibition on the Epac agonist-mediated Rac1 activation. Rac1 activity was measured by pulldown assay. Representative western blots of active Rac1 (Rac1-GTP), total Rac1, and pERK1/2. ECs were treated with MEK inhibitor (U0126; 10 μM) or DMSO for 30 min followed by treatment with 8-CPT (200 μM) for 20 min. ^∗^ vs. control. **(C)** Effect of MEK/ERK inhibition on RhoA/Rock signalling and MLC phosphorylation. ECs were treated with thrombin in the presence of 8-CPT, U0126 (10 μM), U0126 plus 8-CPT, or DMSO as indicated. Representative western blots of MYPT1 phosphorylation at T850 (Rock target) and MLC phosphorylation at S18/T19 (MLCK target). GAPDH was used as loading control. Representative blots from three experiments.

VE-cadherin is anchored on peripheral actin cytoskeleton that strengthens its localisation at the cell-cell junction (Komarova et al., 2017). As shown in Figure 5A, 8-CPT-cAMP enhanced the localisation of actin at cell periphery that was considerably enhanced in the presence of MEK/ERK inhibitor U0126. The MEK inhibitor U0126 alone weakly enhanced the localisation of actin at cell periphery. Small GTPase Rac1 is an important mediator of actin cytoskeleton reorganisation at the cell periphery. Similar to its weak effect on actin localisation, the U0126 alone had a moderate effect on Rac1 activity. On the other hand, the Epac agonist alone caused a three-fold increase in Rac1 activity which, however, was not changed in the presence of MEK/ERK inhibitor (Figure 5B). We further investigated the effect on EC contractile machinery. Under basal conditions, the EC contractile activity is very low, therefore, thrombin was used to induce its activation. Thrombin induced an increased phosphorylation of MLCP regulatory subunit MYPT1 at inhibitory site (T850) which as not abrogated in the presence of either 8-CPT, U0126, or combination of both (Figure 5C). However, thrombin-induced MLC phosphorylation was antagonised by U0126. In the presence of Epac agonist, the thrombin-induced MLC phosphorylation was higher than thrombin alone which was attenuated by MEK inhibitor U0126 (Figure 5C).

### cAMP/Epac Signalling and Endothelial Survival

**FIGURE 6:**
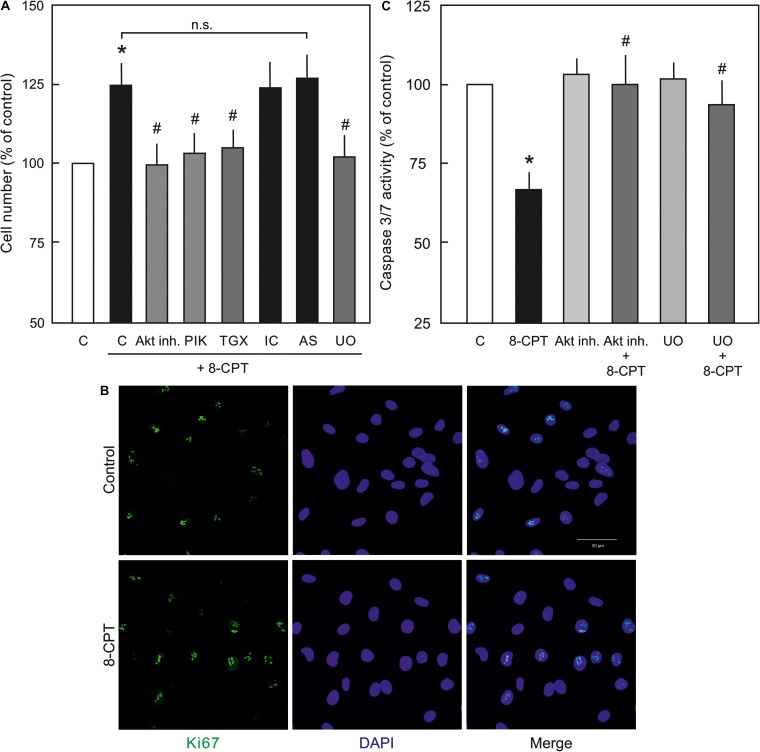
Role of PI3/Akt and MEK/ERK signalling in Epac-mediated EC survival. **(A)** Effect of the Epac agonist on EC cell number. ECs were treated with Akt inh. VIII, isoform specific PI3K inhibitors, MEK inhibitor U0126, or DMSO as indicated for 60 min, followed by removal of the medium containing inhibitors and treated with 8-CPT (200 μM) for 24 h and the cells were counted afterwards. ^∗^ vs. control; # vs. 8-CPT alone; n.s.: not significantly different. **(B)** Representative immunofluorescence images of cell proliferation marker Ki67 expression from three experiments of independent cell preparation. Growth arrested ECs were treated with 8-CPT or vehicle for 24 h in the EC medium containing reduced growth factors. The cells were fixed with 4% PFA and immunostained using specific antibody to Ki67. The nuclei were stained with DAPI (Bar = 50 μm). **(C)** Effect of the Epac agonist on EC caspase 3/7 activity. ECs were treated with Akt inh. VIII, MEK inhibitor U0126, or DMSO as indicated for 60 min in growth factors/serum reduced medium, followed by removal of the medium containing inhibitors and treated with 8-CPT (200 μM) for 24 h in growth factors/serum reduced medium and caspase 3/7 activity was measured using Caspase-Glo kit according to manufacturer’s instructions. ^∗^ vs. control; # vs. 8-CPT-cAMP alone.

Since, PI3K/Akt and MEK/ERK signalling is important in EC survival and proliferation (Cobb, 1999), the effect of the Epac agonist on EC survival and proliferation was analysed. As shown in Figure 6A, indeed the cell number in the Epac agonist-treated cells was significantly higher compared to vehicle-treated cells. This effect was abrogated by inhibitors of MEK/ERK, Akt, or PI3Kα and PI3Kβ but not PI3Kγ or PI3Kδ. In order to investigate whether this enhanced cell number is due to increased cell proliferation or survival, expression of cell-proliferation marker Ki67 and caspase 3/7 activity were analysed. In order to avoid the masking effects of growth factors present in the complete EC culture medium, these experiments were performed under reduced growth factors and serum conditions. As shown in Figure 6B, no significant difference in Ki67 expression between control and Epac-agonist treated cells was observed. However, a ∼35% reduction in caspase 3/7 activity was observed in the Epac-agonist treated cells which was completely abrogated by the inhibition of both PI3K/Akt and MEK/ERK signalling (Figure 6C). In order to avoid the toxicity due to permanent inhibition of Akt or MEK/ERK, the medium containing the inhibitors was replaced with the one without the inhibitors. Their inhibitory effect under these conditions was confirmed by determining the phosphorylation of their targets (Supplementary Figure S3).

## Discussion

The major findings of the present study are: (1) ECs express PI3Kα, PI3Kβ, and PI3Kγ but not PI3Kδ isoforms and cAMP/Epac signalling potently activates PI3K/Akt signalling via activation of PI3Kα and PI3Kβ isoforms, (2) cAMP/Epac signalling activates MEK/ERK pathway and an inhibition of MEK/ERK signalling enhances EC barrier stabilising effects of cAMP/Epac signalling mainly via actin cytoskeleton remodelling, (3) cAMP/Epac signalling promotes EC survival via PI3K/Akt and MEK/ERK pathways.

Endothelial cell barrier integrity is regulated by the actin-myosin based contractile machinery and actin cytoskeleton-anchored AJs consisting of VE-cadherin and catenins directly connected to the actin cytoskeleton (Dejana et al., 2008). We and others have demonstrated that cAMP signalling plays an important role in the regulation and maintenance of EC barrier integrity mainly via modulating the activities of Rho GTPases and EC contractile machinery (Patterson et al., 2000; Waschke et al., 2004; Aslam et al., 2010). Intracellular cAMP downstream activates two effectors PKA and Epac, both of which play an important role in the regulation of EC barrier function but via different signalling mechanisms, which partly converge on Rac1 activation (Aslam et al., 2014; Schlegel and Waschke, 2014).

### Epac and PI3K/Akt Signalling

Activation of cAMP/Epac signalling activates PI3K/Akt signalling pathway in various cell types including ECs (Namkoong et al., 2009; Baviera et al., 2010), but isoform-specific activation of PI3K by Epac is not known. Our mRNA expression data demonstrate for the first time that HUVEC express PI3Kα, PI3Kβ, and PI3Kγ but not PI3Kδ isoforms and cAMP/Epac signalling activates PI3K/Akt pathway mainly via activation of PI3Kα and PI3Kβ isoforms. Interestingly, the expression of PI3K isoforms is relatively lower in the freshly isolated HUVEC compared to passages 1–2. One possible explanation of this may be that the ECs in intact vessel are quiescent while in culture they are more proliferative and thus require higher expression and activity of PI3K. The role of PI3K/Akt signalling in the regulation of EC barrier integrity is controversial and seems to be context-dependent. VEGF and TNFα signalling disrupt EC barrier function via activation of PI3K/Akt signalling (Di et al., 2009; Cain et al., 2010) and tumour cells transmigrate through EC monolayers via induction of PI3K signalling in ECs (Eum et al., 2004). In contrast insulin- and adenosine A1 receptor-mediated activation of PI3K/Akt signalling protects EC barrier function (Gündüz et al., 2010; Umapathy et al., 2013). In the present study, no significant effect of transient inhibition of PI3K/Akt signalling on basal as well as thrombin-induced EC hyperpermeability was observed. Moreover, our data show for the first time, although PI3K/Akt signalling is strongly activated by the Epac agonist, PI3K/Akt signalling plays no significant role in mediating its EC-barrier stabilising effects. It has been shown that in arterial ECs Epac activity is regulated by PDE3B and in particular that PDE3B-based tethering of EPAC1 is critical for EPAC1-mediated activation of PI3Kγ downstream signaling (Wilson et al., 2011). Accordingly, pharmacological inhibition or knockdown of regulatory subunit of PI3Kγ resulted in an inhibition of Epac signalling while PDE3B knockdown resulted in an activation of Epac signalling (Wilson et al., 2011). In the present study, an inhibition of Akt phosphorylation in the presence of PI3Kγ inhibitor was not observed in HUVEC suggesting a cell-type specific regulation of cAMP signalling by PI3Kγ. In contrast we do see a slight increase in phospho-Akt band in PI3Kγ inhibitor treated cells, but did not further investigate in this direction and this question remains open for further investigation.

Interestingly, Wortmannin, a commonly used pan PI3K inhibitor, caused an increase in basal EC permeability and loss of cell-cell junctions (Figure 3) which could not be reproduced with any of the isoform specific inhibitors of PI3K or downstream Akt inhibitor suggesting its non-specific off-target effects such as inhibition of mTOR, MLCK, (Ihle and Powis, 2009) and particularly actin cytoskeleton modulation (Arcaro and Wymann, 1993). Changes in actin cytoskeleton i.e., increased stress fibres and loss of peripheral actin, were observed in the present study (Figure 3B). The Epac agonist could reinstate the actin at cell periphery thus efficiently antagonising Wortmannin-induced loss of EC barrier integrity (Figure 3). The Epac agonist-mediated actin cytoskeleton remodelling is mediated via Rac1 activation (Waschke et al., 2004; Lorenowicz et al., 2008; Spindler et al., 2010).

### Epac and MEK/ERK Signalling

Epac signalling causes an activation of MEK/ERK signalling via Ras-dependent B-Raf activation (Busca et al., 2000; Namkoong et al., 2009). However, the role of MEK/ERK pathway in Epac-mediated EC barrier stabilisation is not studied yet. Several agonists such as thrombin, endothelin, integrins, and activated leukocytes can induce activation of cellular MEK/ERK pathway both in ECs and non-ECs (Nguyen et al., 1999; Mansfield et al., 2000; Haidari et al., 2011) and pharmacological inhibition of MEK/ERK signalling has been shown to protect against inflammatory lung injury (Schuh and Pahl, 2009). The present study demonstrates that the Epac agonist enhanced ERK phosphorylation in ECs and inhibition of MEK/ERK signalling significantly enhanced EC barrier stabilising effect of the Epac agonist and potentiated its protective effect against thrombin-induced hyperpermeability. However, inhibition of MEK/ERK signalling alone had no effect on basal EC permeability or thrombin-induced hyperpermeability. EC cytoskeleton plays a vital role in the maintenance of AJs and barrier integrity (Hartsock and Nelson, 2008). Epac activation alone causes reorganisation of peripheral actin via an activation of Rac1 (Spindler et al., 2010). Rac1 competes with RhoA/Rock signalling which in contrast regulates actin stress fibre formation across the ECs. Cellular GTPase activities and actin cytoskeleton exists in a dynamic form shuttling actin constantly between monomeric (G-actin) and polymeric (F-actin) form and specific temporal activity of a particular GTPase (Rac1 and RhoA) may shift actin from stress fibre to periphery or vice versa. In the present study, the Epac agonist caused a strong activation of Rac1 and enhanced the localisation of actin at cell periphery without affecting stress fibres possible using the available monomeric G-actin. MEK inhibitor alone moderately increased Rac1 activity which probably is not sufficient to reduce the basal permeability. However, in combination with the Epac agonist, a strong shift of actin cytoskeleton from stress fibres to peripheral actin is observed suggesting either RhoA/Rock signalling is inhibited or a speedy and permanent activation of Rac1 shifts the balance towards peripheral actin leading to reduction in stress fibres. We have previously demonstrated that the Epac agonist itself does not antagonise RhoA/Rock signalling (Aslam et al., 2010), and MEK inhibitor alone or in combination with the Epac agonist also did not antagonise RhoA/Rock pathway in ECs (Figure 5C). Therefore, it can be assumed that an enhanced EC barrier protective effect is mediated via actin remodelling via shifting balance towards peripheral actin without affecting RhoA/Rock signalling. Another possible explanation for this additional barrier protection could be that p44/42 MAPK can directly phosphorylate and activate MLC kinase (MLCK) that downstream phosphorylates MLC (Klemke et al., 1997) thereby triggering EC contraction and barrier destabilisation. An inhibition of MEK/ERK pathway thus indirectly inhibits MLCK activation and EC contraction thereby contributing towards EC barrier stabilisation (Kevil et al., 2000; Verin et al., 2000). This is indeed demonstrated in the present study (Figure 5C).

### Epac and EC Survival

PI3K/Akt and MEK/ERK signalling are well-known to play an integral role in EC proliferation (Dayanir et al., 2001; Nakagami et al., 2001). However, effect of cAMP/Epac activation on cell proliferation is contradictive. In smooth muscle cells and fibroblasts Epac activation inhibits cell proliferation *in vitro* (Haag et al., 2008; Kassel et al., 2008; Hewer et al., 2011), while in ECs it induces cell proliferation in an Akt-dependent manner (Namkoong et al., 2009). Accordingly, we observed an increased cell number in the Epac agonist-treated ECs that was abrogated by pharmacological inhibitors of MEK, Akt, and PI3Kα and PI3Kβ but not PI3Kδ or PI3Kγ. In order to distinguish between cell proliferation and survival as the possible cause of the increased cell number, the expression of the cell proliferation marker Ki67 was analysed. No significant change in the expression of Ki67 between control and the Epac agonist-treated cells was observed suggesting increased cell number might not be due to enhanced cell proliferation. In contrast, a strong reduction in caspase 3/7 activity was observed in the Epac agonist-treated cells that was abrogated by inhibition of PI3K/Akt or MEK/ERK signalling. These data confirm that increased cell number is due to enhanced cell survival but not proliferation. A cellular protective effect of Epac signalling has previously been reported in kidney epithelial cells (Qin et al., 2012). However, in contrast Epac activation in neuronal cells and cardiomyocytes has been shown to be pro-apoptotic (Suzuki et al., 2010), suggesting Epac effects are cell-type specific and may vary in different cell types.

## Limitations, Conclusion, and Outlook

**FIGURE 7:**
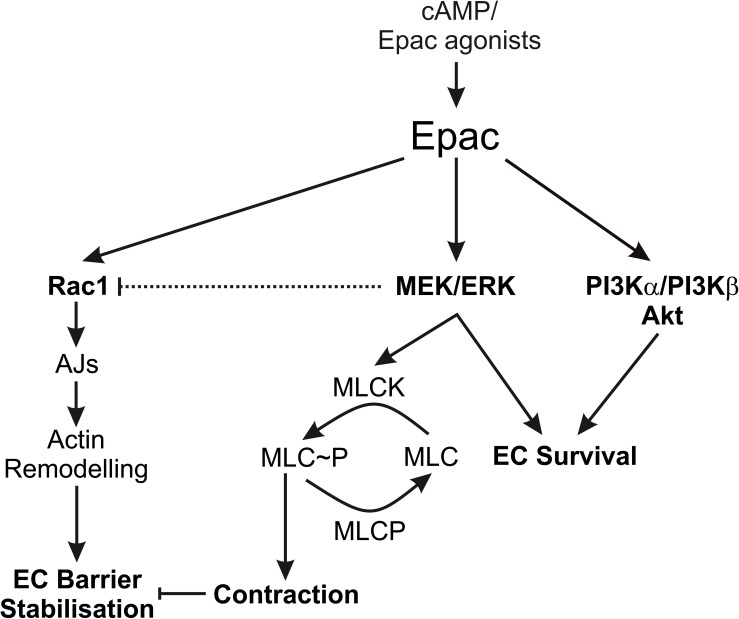
Schematic summary of the data. The scheme summarises the effects of the Epac agonist on EC PI3K/Akt and MEK/ERK signalling in relation to EC barrier function. AJs, Adherens junctions; EC, Endothelial cells; MLC, Myosin light chains; MLCK, MLC kinase; MLCP, MLC phosphatase.

The authors acknowledge the limitations that the study is mainly based on the use of pharmacological inhibitors, but still the data provides some insight about the complex mechanisms of Epac signalling and endothelial barrier function. Particularly, the regulation of Rac1 activity by MEK/ERK signalling is interesting and further investigations may explore the complex interaction of MEK/ERK and Rac1 signalling. Summarising, our data demonstrate that the Epac agonist simultaneously activates diverse signalling pathways that may have differential effects on EC barrier function. It activates PI3K/Akt and MEK/ERK signalling which mainly govern its pro-survival effects on ECs. Inhibition of MEK/ERK but not PI3K/Akt signalling enhances barrier stabilising and barrier protective effects of cAMP/Epac activation. Figure 7 schematically summarises the previously available information and the data from this study.

## Author’s Note

The data from this manuscript was presented in abstract (Poster) form at “Frontiers in Cardiovascular Biology” meeting in April 2018 (Vienna) and “Europhysiology” meeting in September 2018 (London).

## Data Availability Statement

All datasets generated for this study are included in the article/Supplementary Material.

## Ethics Statement

The studies involving human participants were reviewed and approved by Ethics committee of University Hospital of Giessen and Marburg. The patients/participants provided their written informed consent to participate in this study.

## Author Contributions

DG designed the study, performed analysis, and critically reviewed the manuscript. CTr, CTa, SR, and CH performed analysis and critically reviewed the manuscript. MA designed the study, performed experiments, and drafted the manuscript.

## Conflict of Interest

DG and CT were later employed by a private trust hospital “Evangelisches Jung Stilling Krankenhaus GmbH.” The remaining authors declare that the research was conducted in the absence of any commercial or financial relationships that could be construed as a potential conflict of interest.
