# Supplementary material for: Role of PI3K/Akt and MEK/ERK Signalling in cAMP/Epac-Mediated Endothelial Barrier Stabilisation
Source: Front Physiol. 2019 Nov 7;10:1387. doi: 10.3389/fphys.2019.01387 (PMC6855264; doi:10.3389/fphys.2019.01387)
Supplement: Supplementary file 1 [file Data_Sheet_1.PDF]

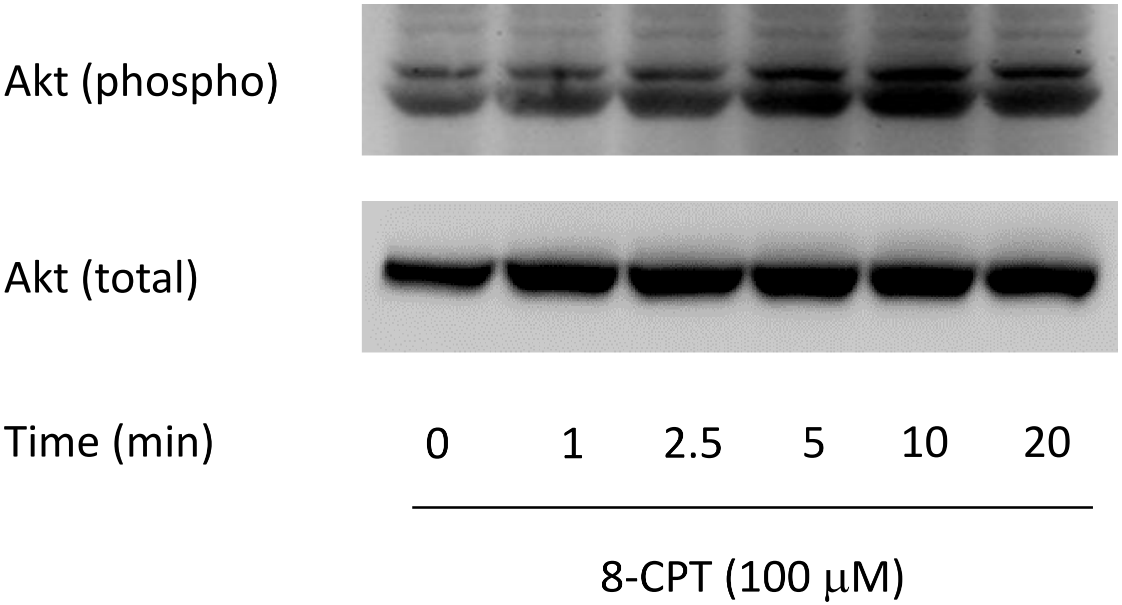

Fig. S1: 8-CPT activates (phosphorylates) Akt.
HUVEC were treated with 8-CPT (100 μM) for different time points as indicated and lysed in laemlli buffer. The membranes were blotted against Akt phospho and total using respective antibodies.

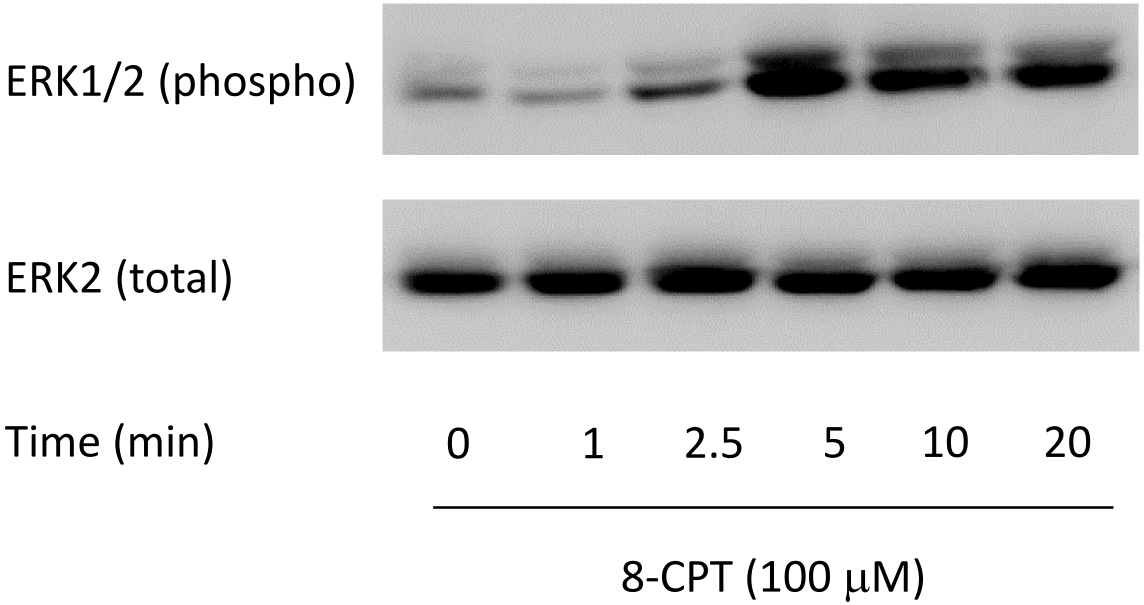

Fig. S2: 8-CPT activates (phosphorylates) MAPK ERK.
HUVEC were treated with 8-CPT (100 μM) for different time points as indicated and lysed in laemlli buffer. The membranes were blotted against ERK1/2 phospho and ERK2 total using respective antibodies.

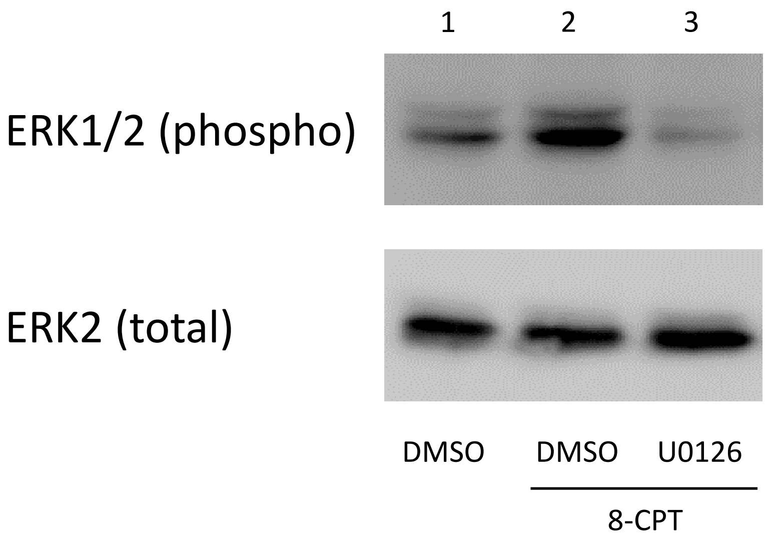

Fig. S3: U0126 and 8-CPT-induced ERK phosphorylation. HUVEC were treated with DMSO (1 and 2) or U0126 (10 μM; 3) for 30 min as indicated. Afterwards, the buffer containing drugs was removed and the cells were treated with 8-CPT (100 μM; 2 and 3) or buffer (control; 1) for 5 min as indicated and lysed in laemlli buffer. The membranes were blotted against ERK1/2 phospho and ERK2 total using respective antibodies.
